# The effects of small geographical resolution and age on the phyllosphere microbial diversity of *Castanopsis eyrei* in subtropical forest

**DOI:** 10.1128/spectrum.02091-24

**Published:** 2025-02-12

**Authors:** Lei Xie, XuXu Bao, Shuifei Chen, Hui Ding, Yanming Fang

**Affiliations:** 1 Co-Innovation Center for Sustainable Forestry in Southern China, College of Life Sciences, Key Laboratory of State Forestry and Grassland Administration on Subtropical Forest Biodiversity Conservation, Nanjing Normal University 224704 , Nanjing, Jiangsu, China; 2 Department of Biological Sciences, National University of Singapore, , Singapore; 3 Research Center for Biodiversity Conservation and Biosafety, State Environmental Protection Scientific Observation and Research Station for Ecological Environment of Wuyi Mountains, Biodiversity Comprehensive Observation Station for Wuyi Mountains, State Environmental Protection Key Laboratory on Biosafety, Nanjing Institute of Environmental Sciences, Ministry of Ecology and Environment of China, Nanjing, China; University of the Philippines Los Baños, Laguna, Philippines

**Keywords:** *Castanopsis eyrei*, third-generation full-length sequencing, microbial diversity, phyllosphere, community assembly

## Abstract

Our understanding of the mechanisms that maintain phyllosphere microbial diversity in natural systems remains far less developed than our understanding of belowground microbiomes. This knowledge gap hinders our comprehension of growth dynamics in the Fagaceae, a predominant tree family in subtropical regions, and the critical role it plays as a major forest community assembly. Here, we tested leaves from *Castanopsis eyrei*, a widespread subtropical Fagaceae species in south-eastern China, sampled across multiple ages within a forest dynamics plot on Mt. Huangshan. Using third-generation sequencing of full-length bacterial 16S rRNA genes and fungal ITS regions, we characterized the associated phyllosphere microbiota. We found that phyllosphere fungal diversity was high, dominated by Teratosphaeriaceae, Trimorphomycetaceae, and Bulleribasidiaceae, while bacterial diversity was lower and primarily comprised Beijerinckiaceae, Isosphaeraceae, and Acidobacteriaceae. Habitat, rather than host age, emerged as the principal factor influencing fungal and pathogen diversity. Linear mixed-effects models revealed a negative relationship between *C. eyrei* biomass and phyllosphere pathogen diversity. Co-occurrence network analysis showed that *C. eyrei* saplings supported the most complex network structure, with *Recuromyces* acting as a key pathogenic fungus, serving as both a module hub and a connector across all age classes. Overall, these findings highlight the ecological importance of habitat in shaping phyllosphere microbial diversity and underscore the interplay between host function and the maintenance of microbial diversity.

**IMPORTANCE:**

Plant surfaces host diverse microbial communities that significantly impact host health and overall forest productivity. However, mechanisms maintaining phyllosphere microbial diversity and their consequences for host plants remain poorly understood. Employing a three-generation high-throughput sequencing approach, we investigated the phyllosphere fungal and bacterial diversity across different microhabitats and ages of *Castanopsis eyrei*, a common species in subtropical forest, China. Our results underscore the presence of exceptionally high microbial diversity on the plant surface, elucidating the taxonomic composition at the family level of key host microorganisms. Furthermore, our observation of a negative correlation between host performance and phyllosphere pathogens underscores the potential self-limiting ability of plants.

## INTRODUCTION

The combined surface area of all plant leaves on Earth is estimated to encompass roughly 109 km^2^, an area approximately twice that of the Earth’s surface (1). This large leaf surface serves as one of the largest microbial habitats globally, underscoring its significance. Within this context, the phyllosphere—referring to the entire living leaf structure and encompassing the epidermal cells alongside the upper and lower leaf surfaces—acts as a habitat for an array of microorganisms (2). These microbes colonize the leaf’s surface as epiphytes, reside within its cells as endophytes, or act as phytopathogens (3). Aboveground phytopathogenic fungi could influence host plants by infecting leaves, thereby impacting plant photosynthesis and respiration and ultimately influencing plant fitness (4, 5). Nonetheless, our comprehension of phyllosphere microbial diversity and its impacts significantly lag behind that of belowground microbial diversity. Notably, microorganisms exhibit pronounced convergence at higher taxonomic levels while revealing significant differences at finer taxonomic resolutions among various plant species (6). The results of 454 pyrosequencing and second-generation high-throughput sequencing focus on the phylum level, further limiting our understanding of the intricate interplay among the community components within the phyllosphere microbial diversity.

As a pivotal component of the plant microbiome, the microorganisms of the phyllosphere wield the potential to impact plant species’ coexistence through stabilizing mechanisms and equalizing mechanisms (5). Notably, the presence of pathogenic fungi within the phyllosphere environment can lead to the transmission of diseases to susceptible neighboring plants (7). This, in turn, exerts an influence on plant population structure, subsequently affecting the dynamics of plant communities. Moreover, the Janzen-Connell hypothesis posits that organisms such as pathogenic fungi and herbivores can curtail the renewal and growth rates of adjacent mature individuals of the same plant species, causing a negative density-dependent effect (8, 9). This process, in effect, creates space and resource availability for other species, thereby fostering species coexistence. It emphasizes that plants possess self-limiting capabilities (10). However, research to date has primarily concentrated on the impact of soil-borne pathogens on plant self-limitation, with limited studies exploring the effects of phyllosphere pathogens on plant self-limitation. A detailed understanding of the interplay between plant performance and the diversity of phyllosphere pathogenic fungi is therefore of pivotal importance. As a consequence, our hypothesis, tested in the current study, posits that phyllosphere pathogenic fungi could play a role in fostering plant species coexistence through stabilization mechanisms, potentially manifested in the correlation between the diversity of leaf pathogenic fungi and plant functional traits.

Tree species in the Fagaceae family hold a significant position in subtropical forest community assemblages (11). Hence, understanding the diversity of microorganisms associated with these tree species is crucial for comprehending the self-limiting capacity of Fagaceae plants. Currently, 454 pyrosequencing and second-generation high-throughput sequencing have emerged as the predominant methodologies for acquiring data associated with microbial diversity (12, 13). Notably, studies on fungal communities within temperate oak-based ecosystems have highlighted their diversity and revealed distinct variations between trees inhabiting urban and non-urban settings (2, 14). However, the use of third-generation full-length sequencing, coupled with its high-resolution taxonomic capabilities, remains somewhat limited in the realm of plant-based diversity assessment. Consequently, our understanding of the fundamental phyllosphere microbial taxa is still inadequate. Recognizing the substantial influence of phyllosphere microorganisms on plant growth and the intricate interplay between phyllosphere fungi and plant performance, our study sought to leverage third-generation sequencing. By doing so, our goal was to reveal the intricate tapestry of phyllosphere microbial diversity and the potential mechanisms driving its variation.

Presently, the mechanisms governing the maintenance of microbial diversity in the phyllosphere are a subject of active research. Existing perspectives propose that factors such as plant age, host species identity and genotype, geographic location, and temporal variation represent the primary influencers of phyllosphere microbial diversity (15–18). However, different viewpoints have emerged from these studies when quantifying the impacts of these biotic and abiotic factors on the *α*- and *β*-diversity of phyllosphere microbes. This divergence underscores the complex and dynamic nature of the sources that contribute to the phyllosphere microbial pool. On one hand, it is imperative to embrace a holistic approach to field observation experiments to comprehensively identify the potential influences on phyllosphere microbial diversity. On the other hand, while previous research has predominantly focused on bacterial diversity, the equally important realm of fungal diversity has often been overlooked. Moreover, it is crucial to recognize that the mechanisms governing the maintenance of the diversity of endophytic and exophytic microorganisms may exhibit differences. Notably, an experimental study within a subtropical forest ecosystem in Southeastern China, investigating forest biodiversity and ecosystem function (BEF), revealed clear structural differences between leaf-associated and endophytic bacterial communities (19). This study indicated that bacterial species diversity within the forest, along with the identity and traits of the host plant, had a more pronounced influence on the endophytic than on the epiphytic microbial community. This suggests that leaf epi- and endophytic bacterial communities are constructed by different mechanisms, potentially linked to the intricate interplay between the internal and external leaf environments.

Although previous studies have provided insights into the biogeographic patterns associated with phyllosphere microbial composition and the underlying drivers, a comprehensive assessment encompassing both fungi and bacteria within the same tree species and site has remained less studied (17, 20, 21). Furthermore, the majority of these investigations have predominantly focused on temperate zones, leaving a critical knowledge gap in understanding subtropical ecosystems. In a ground-breaking study, Wang et al. (22) validated the latent impact of tree species on the diversity and stability of phyllosphere bacterial communities in subtropical forests, underscoring the imperative for further investigations into phyllosphere dynamics. The origins of phyllosphere microorganisms are multifaceted and dynamic, influenced by intrinsic plant factors and environmental dynamics. Simultaneously, the interplay of neutral and niche processes must be considered to interpret the entire picture (23, 24). The subtropical climatic backdrop provides an ideal breeding ground for diverse microbial species, rendering it to be a compelling locale for unraveling the intricacies of phyllosphere microbial diversity. This study is uniquely poised to analyze the ecological drivers that shape the composition of phyllosphere microbial communities within a single subtropical tree species, taking into account the multifaceted influences of numerous drivers. The primary objectives of this study are threefold: (i) to profile the phyllosphere microbiome associated with the dominant tree species of subtropical forests; (ii) to determine the impacts of varied habitats and tree ages on phyllosphere microbial community composition, probing potential factors influencing their diversity; and (iii) to uncover patterns of correlation linking host plant performance with the microbial community diversity in the phyllosphere.

## MATERIALS AND METHODS

### Study site and sample collecting

Situated in Southeastern China, Mount Huangshan boasts a subtropical monsoon climate, occupying a pivotal position in the mid-latitude global spectrum and experiencing distinct seasons (11). In 2014, a 10.24 hm^2^ forest dynamics plot was established at the western part of Mount Huangshan in Xiaolingjiao (XLJ), Anhui Province, to investigate the community dynamics of the forest. The plot was situated at an elevation range of 400–600 m. XLJ contains 170 species of woody plants, belonging to 56 families and 110 genera. We used multiple regression trees (MRT) to divide the XLJ into three habitats. The employed habitat classification approach, integrating both plot topographic factors and community species distribution, finds extensive application in forest dynamics plots. *Castanopsis eyrei* (Champ. ex Benth.) Hutch. was the species with the highest importance value in each habitat and the highest importance value (23.29%) over the whole sample site (25). In addition, *C. eyrei* is one of the most common conifer-broadleaf tree species in the subtropical regions of China (26). It plays a vital role in carbon balance within subtropical forest ecosystems (27). Therefore, in this study, we selected *C. eyrei* as our study species.

Our field survey was conducted in September 2022. The diameter at breast height (DBH) is commonly used as a proxy for estimating *C. eyrei* tree age, with larger DBH generally indicating older individuals compared with smaller ones. To classify the trees in our study, we employed the 99% quantile of each species’ DBH (DBH99) as a criterion. Individuals with DBH ＜ DBH99^1/2^ were categorized as saplings, while those with DBH ≥ DBH99^2/3^ were considered adults. The range between DBH99^1/2^ and DBH99^2/3^ was not precisely divided to ensure accuracy in the different diameter classes (28). Although these classifications do not strictly represent small (saplings) trees and adult trees, this approach simplifies the analysis. Applying this classification method, we defined *C. eyrei* young trees as those with 1 cm <DBH ≤ 4.0 cm (Saplings, YT), middle-aged trees as those with 4.0 cm <DBH ≤ 6.7 cm (Juveniles, MT), and adult trees as those with DBH >6.8 cm (Adults, MAT). Using these three diameter thresholds, we aimed to collect phyllosphere samples of *C. eyrei* from distinct age classes.

Furthermore, to consider the effect of habitat on phyllosphere microbial diversity, we selected phyllosphere samples from each habitat in the plot site from different ages of *C. eyrei* trees. The three habitats were as follows: habitat A, low-elevation ridges; habitat B, valley habitats; and habitat C, high-elevation ridges. We selected the number of samples from each habitat proportionately based on the area of each of the three habitats, resulting in five samples from each of habitats A and C, and six in habitat B, for a total of sixteen 20 m × 20 m quadrats (Fig. S1). In each 20 m × 20 m quadrat, three subsamples were collected from each of the three distinct age groups (YT, MT, and MAT) of *C. eyrei*. Using sterilized scissors, 60–80 leaf samples were collected from around the canopy and pooled together in a sterile bag. Finally, we obtained a total of 48 fresh phyllosphere samples of *C. eyrei*. Two to three sterile medical cotton swabs, moistened with buffer solution, were used to wipe the leaf surfaces. The swabs were then agitated in a centrifuge tube containing potassium phosphate buffer solution, centrifuged, the supernatant decanted away, and the pellet resuspended in the buffer solution (1 mL) for preservation.

### DNA extraction and sequencing

DNA extraction was performed on the phyllosphere samples of *C. eyrei*, using the Plant DNA Extraction Mini Kit B (Mabio), following the manufacturer’s instructions. Subsequently, the concentration and purity of the extracted DNA were measured using a NanoDrop One spectrophotometer (Thermo Fisher Scientific). To amplify the full-length 16S rRNA gene and internal transcribed spacer (ITS) sequence, specific primers were utilized. The primer sequences used, with the respective barcodes, were as follows: 27F (AGRGTTYGATYMTGGCTCAG), 1492R (RGYTACCTTGTTACGACTT) for the 16S rRNA gene, and ITS1F (5′-CTTGGTCATTTAGAGGAAGTAA-3′) and ITS4R (5′-TCCTCCGCTTATTGATATGC-3′) for the ITS sequence. The amplification process was carried out using the BioRad S1000 PCR instrument (Bio-Rad Laboratory). The length and concentration of the PCR products were determined using 1% agarose gel electrophoresis. Following electrophoresis, the PCR products were combined in equidensity ratios based on the GeneTools Analysis Software (version 4.03.05.0, SynGene). Subsequently, the mixture of PCR products underwent purification using the HiPure Gel Pure DNA Mini Kit. The sequencing library was obtained according to 16S Amplification SMRTbell® Library Preparation workflow and ITS/unite/v8.0, and the library was sequenced on a PacBio Sequel II platform (Guangdong Magigene Biotechnology Co., Ltd. Guangzhou, China).

### Bioinformatics analyses

The PacBio data obtained were processed using SMRT Link software (version 6.0). The processing steps included data splitting, sequence error correction, and sequence format conversion, resulting in the generation of clean data. To classify the sequences into operational taxonomic units (OTUs), a 97% similarity threshold was applied using the UPARSE algorithm from the USEARCH software (V10, http://www.drive5.com/usearch/). During the clustering process, UPARSE was able to simultaneously eliminate chimeral sequences and singleton OTUs. The taxonomic information for each representative sequence was assigned by mapping against the SILVA database (v132, https://www.arb-silva.de/) to facilitate the annotation process. We employed FUNGuild (http://www.funguild.org/) to discriminate between fungal trophic modes and functional guilds. We specifically retained the classifications of “highly probable” or “probable” to prevent excessive interpretation of the fungal functional guilds.

### Leaf function, biomass, and topographic factors

Leaf samples were subjected to assays to determine nine functional traits, namely leaf area (LA), specific leaf area (SLA), leaf dry matter content (LDMC), fresh weight (FW), dry weight (DW), leaf organic carbon (LOC), total nitrogen (TN), total phosphorus (TP), and pH. For each tree, measurements were obtained based on 10 leaves, and the mean value was calculated. Leaf area (LA) was estimated by scanning the leaves and determining the size of the digital images using Adobe Photoshop CS6. Fresh weight (FW) was measured by weighing the fresh leaves, while dry weight (DW) was determined by weighing the leaves after oven-drying them at 80°C for 48 hours. LDMC was calculated as the ratio of leaf dry weight to fresh weight, while SLA was calculated as the ratio of leaf area to dry mass. These five traits represented leaf morphology.

The four remaining leaf traits represented leaf physiology. Leaf organic carbon (LOC) and total nitrogen (TN) concentrations were determined using an elemental analyzer (2400 II CHNS; Perkin-Elmer). The leaf total phosphorus (TP) concentration was determined using the molybdate/ascorbic acid method after digestion with H_2_SO_4_–H_2_O_2_. For leaf pH measurements, a 3.00 ± 0.01 g fresh weight subsample of healthy mature leaves, was collected from the sunny side of each *C. eyrei* tree. The number of leaves collected ranged from 20 to 40, depending on the mean leaf size. The collected leaf samples were promptly frozen and then ground with a mortar and pestle. The macerated leaves were then transferred to a 50 mL centrifuge tube with 30.00 ± 0.10 g of deionized water, resulting in a leaf-to-water mass ratio in the tube of 1:10 (29).

In this study, we utilized the methods described by Gonzalez‐Akre et al. (30) to measure aboveground biomass. These methods incorporate a comprehensive and up-to-date database of allometric equations. By incorporating factors such as sample size, climate, and taxonomy, the equations were weighted to account for variation and enhance the accuracy of aboveground biomass calculations. The specific allometric equations (1) are as follows:


 (1) $$AGB=\;10^{-0.77107+2.1534\times \left(ln^{DBH}\right)}$$

where DBH refers to diameter at breast height and AGB refers to aboveground biomass. The CTFS (Center for Tropical Forest Science) approach was employed to calculate four topographic factors and has been used by other researchers (31).

### Quantification of species diversity and phylogenetic diversity

Both fungal and bacterial diversity were assessed in the 48 phyllosphere samples of *C. eyrei* collected from 16 quadrats. Species diversity was measured using various indices including richness, Shannon diversity (SD), and Simpson diversity (SiD). However, it should be noted that the Shannon index and Simpson index do not fully comply with the replication principle (32). To address this issue, we applied an exponential transformation to the Shannon and Simpson indices. Faith’s phylogenetic diversity index (PD) was used to measure fungal and bacterial phylogenetic diversity. Because the bacterial diversity index did not conform to a normal distribution (Shapiro test, *P* < 0.05), we employed the Kruskal-Wallis test to evaluate whether there were significant differences in bacterial diversity among the different habitats. For fungal diversity, which approximated a normal distribution, we used ANOVA to determine whether there were significant differences observed among the three habitats.

### Data analysis

All statistical analyses were performed using R version 4.2.3 (33). To examine the effects of habitats and *C. eyrei* tree age on microbial diversity, an analysis of variance (ANOVA) was conducted. To identify community compositions that were not influenced by variations in richness, pairwise modified Raup–Crick dissimilarity matrices were computed for pathogenic fungi, total fungi, and total bacteria groups, following the approach outlined by Chase et al. (34). Permutational analysis of variance (PERMANOVA) was subsequently employed to investigate differences in community compositions among the three habitat and three age categories for the pathogen, total fungal, and bacterial groups. The analysis was conducted using the ADONIS command available in the “vegan” package of R. To understand correlations between the variables geographic distance, leaf morphology, topography, and leaf physiology and the composition of pathogenic, total fungal, and bacterial microbial communities, distance matrices of these variables were subjected to multiple regression of distance matrices (MRM) in the “ecodist package” of R (35), with forward-selection until *p*_adj_ <0.05 for all variables.

We employed Random Forests (RF) as a statistical approach to examine the relationships between various factors, including tree age, habitats, leaf functional traits, and topography, and diversity parameters such as phyllosphere pathogenic, fungal, and bacterial species diversity, as well as phylogenetic diversity. We configured the RF model with 1,000 decision trees and applied a random seed for reproducibility. After an initial analysis of the model results, we conducted a reassessment of the number of trees. To determine the optimal number of trees for the model, we utilized a criterion based on the corresponding abscissa where the curve became flat or exhibited smaller fluctuations. This value was considered to be the optimal tree size, and we replaced the initial set number of trees with this value to achieve a lower out-of-the-bag (OOB) error rate. In addition, we employed cross-validation to enhance the model accuracy by further screening variables and rebuilding the model. The “randomForest” package in R was utilized for model fitting, while the “rfPermute” package was employed for conducting significance testing of the independent variables.

In our study, we investigated the potential relationships between phyllosphere pathogenic species diversity and *C. eyrei* aboveground biomass using the following model (2):


 (2) $$y=Bx+\pi_{habitat}+\varepsilon$$

where *y* refers to the phyllosphere pathogen species diversity; B refers to the aboveground biomass of *C. eyrei*; π is the random factor to account for spatial autocorrelations within each habitat; and ε is the random sampling error. To analyze this relationship, we performed a linear mixed-effects analysis using restricted maximum likelihood estimation. The “lme4” package (36) in R was used for this analysis.

To investigate the potential symbiotic relationships among pathogenic fungi in the phyllosphere of different stages of *C. eyrei* and identify core pathogenic fungi, we used the SparCC to construct a microbial network, inferring co-occurrence between species. The SparCC method, with default parameters, was applied to the filtered table for inter-domain paired correlation calculations (37). Non-significant correlations in the matrix were filtered at a threshold of 0.3 and a significance level of 0.05. Core pathogenic fungi in the phyllosphere of *C. eyrei* were identified based on Zi-Pi network hub nodes. This analysis was conducted using the R package microeco in combination with SpiecEasi (38).

## RESULTS

### Characteristics of phyllosphere microbial α-diversity

**Fig 1:**
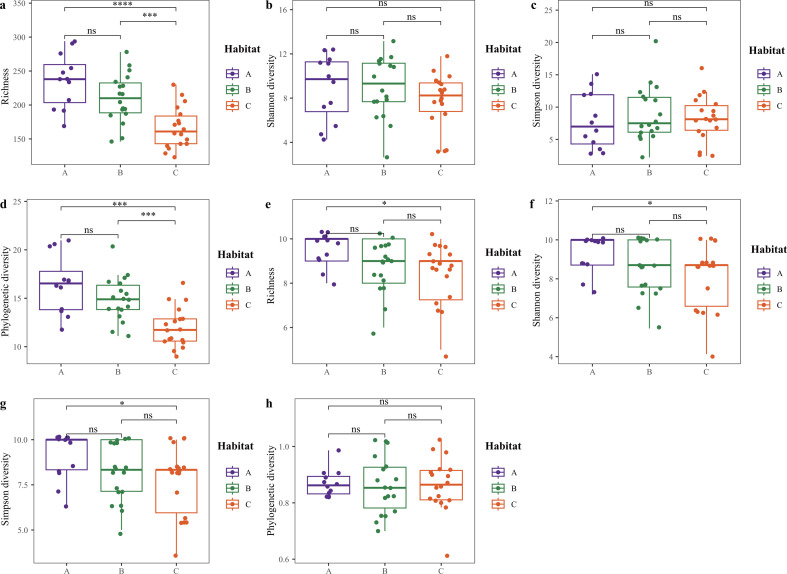
The observed operational taxonomic unit (OTU) richness, Shannon diversity, Simpson diversity, and phylogenetic diversity indices for fungi (a–d) and bacteria (e–h) across the phyllosphere of *C. eyrei*. The line inside each box represents the median value (*n* = 48). Kruskal–Wallis test (e–g) and *t*-test (a–f, and h) were used to test whether significant differences occurred between the two habitats. ns: not significant. *P < 0.05, ***P < 0.01, ****P < 0.001. A: Habitat A, low-elevation ridges; B: Habitat B, valley habitats; C: Habitat C, high-elevation ridges.

**Fig 2:**
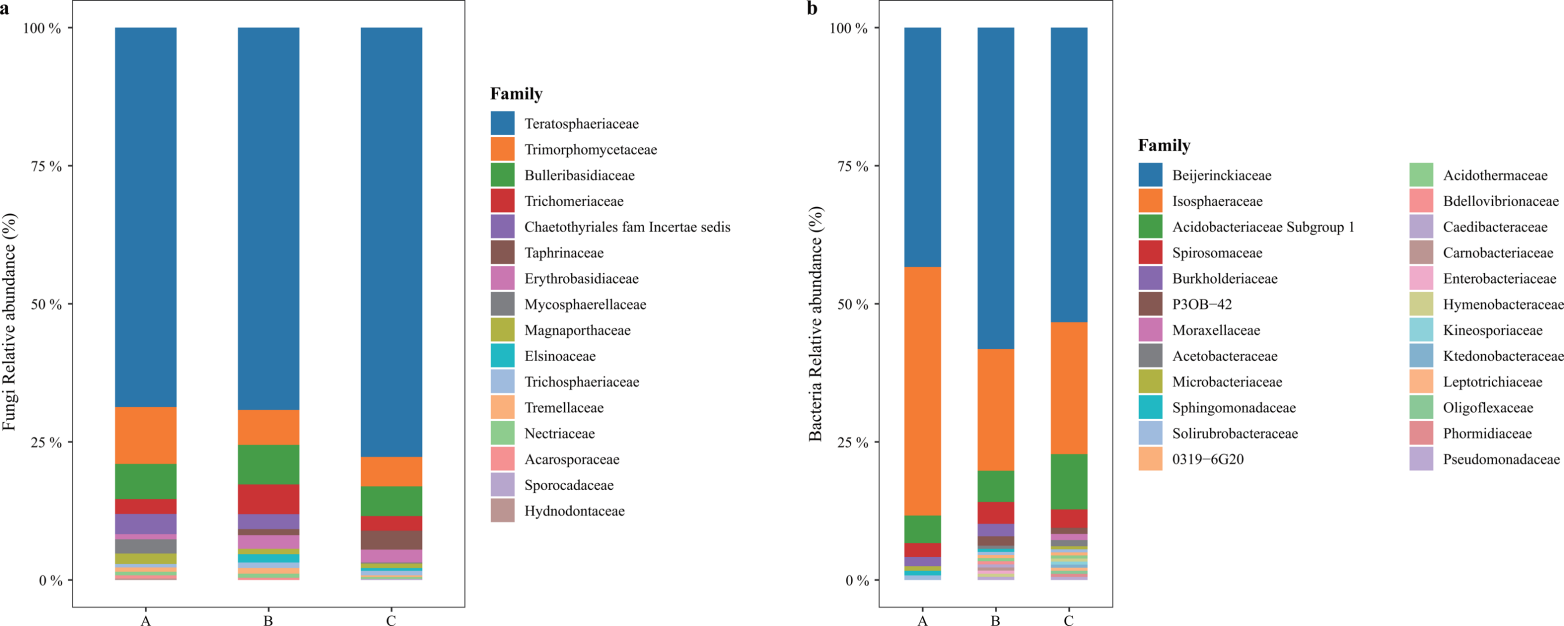
Main families of the *C. eyrei* phyllosphere fungal (a) and bacterial (b) communities in the three habitats, A, B, and C. Here, we removed the unassigned and unclassified taxa.

The results indicated that there were differences between the species diversity of bacteria and fungi in the phyllosphere of *C. eyrei*. The mean ± SD fungal species richness, Shannon diversity, Simpson diversity, and phylogenetic diversity were 200.17 ± 44.47, 2.88 ± 0.50, 8.42 ± 3.96, and 14.20 ± 2.97, respectively. The bacterial species richness, Shannon diversity, Simpson diversity, and phylogenetic diversity were 8.83 ± 1.21, 8.54 ± 1.46, 8.23 ± 1.70, and 0.86 ± 0.09, respectively. The results indicate significant differences in fungal and bacterial diversity between habitats A and C (Fig. 1a, e through g). There were differences in fungal phylogenetic diversity between habitats A and C and between habitats B and C (Fig. 1d). Teratosphaeriaceae, Trimorphomycetaceae, and Bulleribasidiaceae were the most abundant families Fig. 2. The diversity of phyllosphere pathogens varied significantly between habitats A and C and between habitats B and C (Fig. S2 and S3).

In terms of taxonomic classification, we detected a total of seven phyla, 25 classes, 58 orders, 125 families, 149 genera, and 1,720 Operational Taxonomic Units (OTUs) for *C. eyrei* phyllosphere fungi. For phyllosphere bacteria, 17 phyla, 60 orders, 59 classes, 82 families, 100 genera, and 881 OTUs were identified. For the phyllosphere fungi, the dominant families were Teratosphaeriaceae (mean abundance 21.46%), Trimorphomycetaceae (mean abundance 2.06%), and Bulleribasidiaceae (mean abundance 1.80%). As for bacteria, the dominant families were Beijerinckiaceae (mean abundance 50.24%), Isosphaeraceae (mean abundance 25.76%), and Acidobacteriaceae (mean abundance 4.76%) (Fig. 2). At the phylum level, we observed the dominance of the Ascomycota and Basidiomycota phyla in fungi, and the Proteobacteria, Planctomycetes, and Acidobacteria phyla in bacteria (Fig. S4). This study also employed third-generation sequencing technology to elucidate the taxonomic richness of phyllosphere microorganisms at the genus level across different ages of *C. eyrei* trees. The results revealed that the two most abundant genera in bacteria were 1174–901-12 (mean abundance: 47.08%) and *Singulisphaera* (mean abundance: 27.92%) (Fig. S5a). In fungi, the two most abundant genera were *Recurvomyces* (mean abundance: 21.91%) and *Carlosrosaea* (mean abundance: 1.27%) (Fig. S5b). In addition, we observed a substantial proportion of unidentified species at the fungal genus level, exceeding 60% of the total.

Age appeared to have a small significant effect on fungal species diversity and phylogenetic diversity (PD), as indicated in Table 1. However, no significant effects of age were observed between PD and either phyllosphere pathogenic fungal species diversity or bacterial species diversity (Table 1). Significant effects of habitat were observed on phyllosphere pathogenic fungal and fungal species diversity and PD, but there was no significant effect on bacterial diversity (Table 1).

**TABLE 1:** Summary of statistics (F-values and *P* values) from ANOVA on effects of habitats and age on *α*-diversity of phyllosphere of *C. eyrei*^*a*^

Microbiome	Diversity index	Habitat			Age			Habitat ×		
		df	F value	*P*	df	F value	*P*	df	F value	*P*
Phyllosphere pathogen	Shannon diversity	2	13.95	***	2	3.16	0.05	4	2.89	*
	Richness (log-transformed)	2	13.71	***	2	2.93	0.07	4	0.53	0.72
	Simpson diversity (log-transformed)	2	8.14	**	2	1.90	0.16	4	1.99	0.12
	Phylogenetic diversity	2	11.21	***	2	3.29	*	4	0.37	0.83
Fungi	Shannon diversity (square transformation)	2	1.20	0.32	2	3.21	0.05	4	0.32	0.86
	Richness	2	17.41	***	2	4.25	*	4	0.87	0.49
	Simpson diversity	2	0.30	0.74	2	0.29	0.29	4	0.62	0.65
	Phylogenetic diversity	2	15.32	***	2	4.01	*	4	0.97	0.43
Bacteria	Shannon diversity	2	/	0.16	2	/	0.65	4	/	0.82
	Richness	2	/	0.15	2	/	0.62	4	/	0.87
	Simpson diversity	2	/	0.16	2	/	0.68	4	/	0.77
	Phylogenetic diversity	2	0.02	0.98	2	0.69	0.51	4	0.59	0.67

^*a*^ Statistics describe linear regression models of fungal and pathogenic species diversity indices in the phyllosphere of *C. eyrei* while the generalized linear model with gamma error was used to explain the effects of habitat and age on bacterial *α*-diversity. Habitat × Age, the interaction effect between Habitat and Age. ***P < 0.001; **P < 0.01; *P < 0.05. /, bacterial *α*-diversity did not conform to a normal distribution; therefore, the chi-square test was employed for the ANOVA, resulting in no F-value.

### Patterns in phyllosphere microbial β-diversity

The results of PERMANOVA show that the compositions of the pathogen (R = 0.2, *P* < 0.001) and fungal (R = 0.19, *P* < 0.001) communities were significantly different among the three habitats (Table 2). However, the composition of the bacterial community did not vary significantly across the three habitats. Age was not the main factor driving community differentiation (Table 2). Multiple regressions on distance matrices (MRM) were used to further determine the relative contributions of leaf functions, environmental factors, and geographical distance to the similarities among pathogenic, fungal, and bacterial communities. Leaf morphology (LM) traits such as leaf area, dry weight, specific leaf area, and leaf dry matter content had little effect on fungal community composition. The results revealed a positive correlation between geographic distance, leaf morphology (LM) traits, and the similarity in bacterial community composition (Table 3).

**TABLE 2:** PERMANOVA reveals differences in community compositions of groups of pathogens, total fungi, and bacteria among three habitats and ages

Microbiome	Habitat		Age	
	R	P	R	*P*
Pathogen	0.20	<0.001	−0.04	0.92
Fungi	0.19	<0.001	0.02	0.19
Bacteria	0.04	<0.10	−0.01	0.65

**TABLE 3:** Multiple regressions on distance matrices (MRM) of phyllosphere pathogenic, fungal, and bacterial community compositions as predicted by geographic distance (GD), leaf morphology (including LA, DW, SLA, and LDMC), topography, and leaf physiology (including pH, TN, TP, C: N, N: P)^*a*^

Microbiome	GD			Leaf morphology			Topography			Leaf physiology		
	Coef	R^2^ %	*P*	Coef	R^2^ %	*P*	Coef	R^2^ %	*P*	Coef	R^2^ %	*P*
Pathogen	−0.01	NS	NS	0.03	1.57	0.098	0.004	0.06	0.910	0.01	0.83	0.320
Fungi	−0.01	NS	NS	0.02	2.60	**0.035**	0.001	0.41	0.503	0.01	1.09	0.320
Bacteria	0.01	1.47	**0.022**	0.01	1.35	**0.049**	−0.001	NS	NS	−0.002	NS	NS

^*a*^ Boldface indicates statistically significant.

### Factors shaping phyllosphere microbial diversity

**Fig 3:**
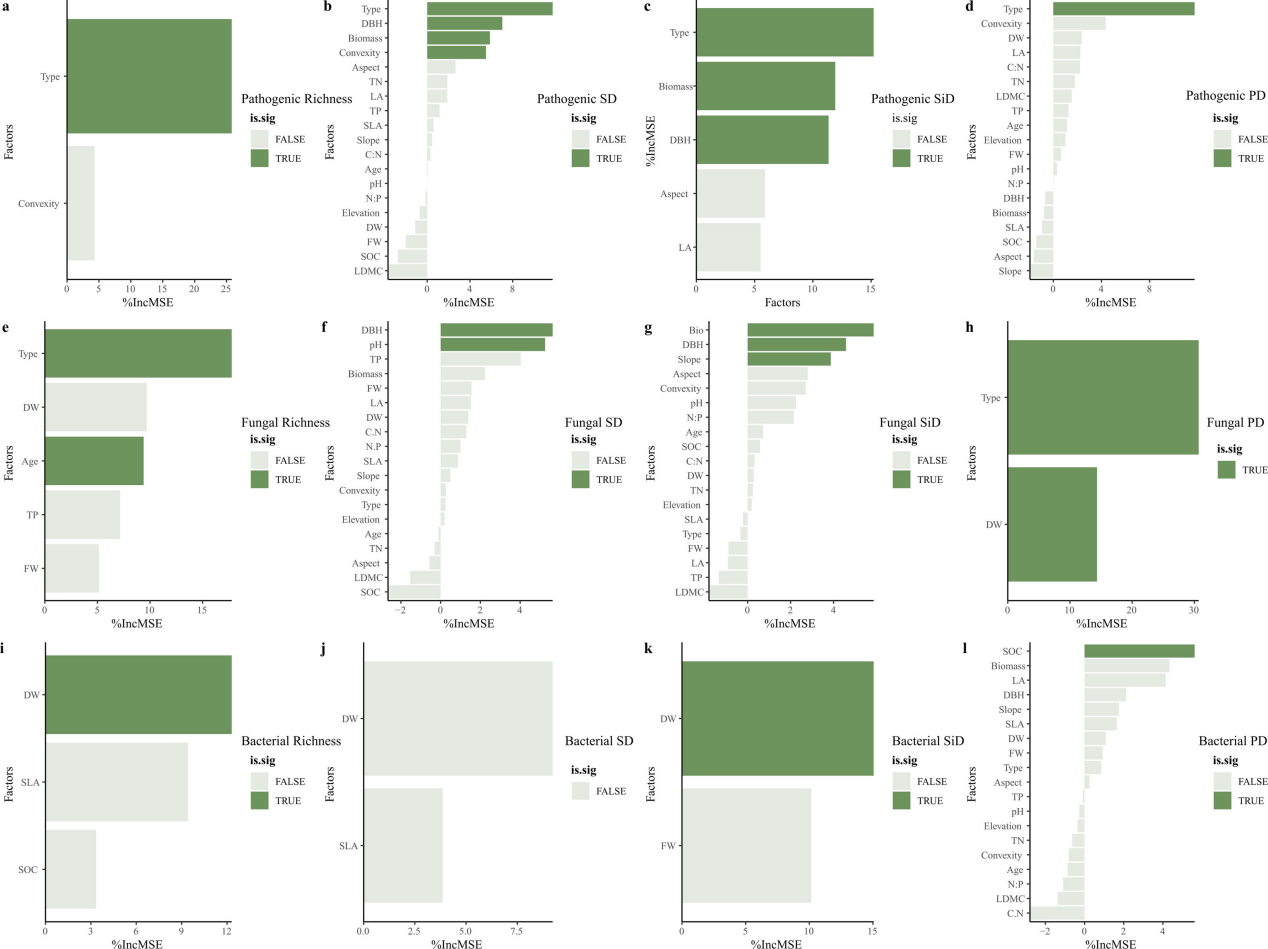
Contributions of factors (age, habitat, leaf function traits, and topography) to the phyllosphere pathogenic (a–d), fungal (e–h), and bacterial (i–l) species diversity and phylogenetic diversity (d**, h, l**). We identified the major significant predictors (marked in green). %IncMSE, the percentage increases in the mean squared error. SD, Shannon diversity, SiD, Simpson diversity, PD, phylogenetic diversity. Note: In the Materials and Methods section, the abbreviations for all factors and references are provided. The highlighted portion, marked in green, is considered to have a significant effect.

**Fig 4:**
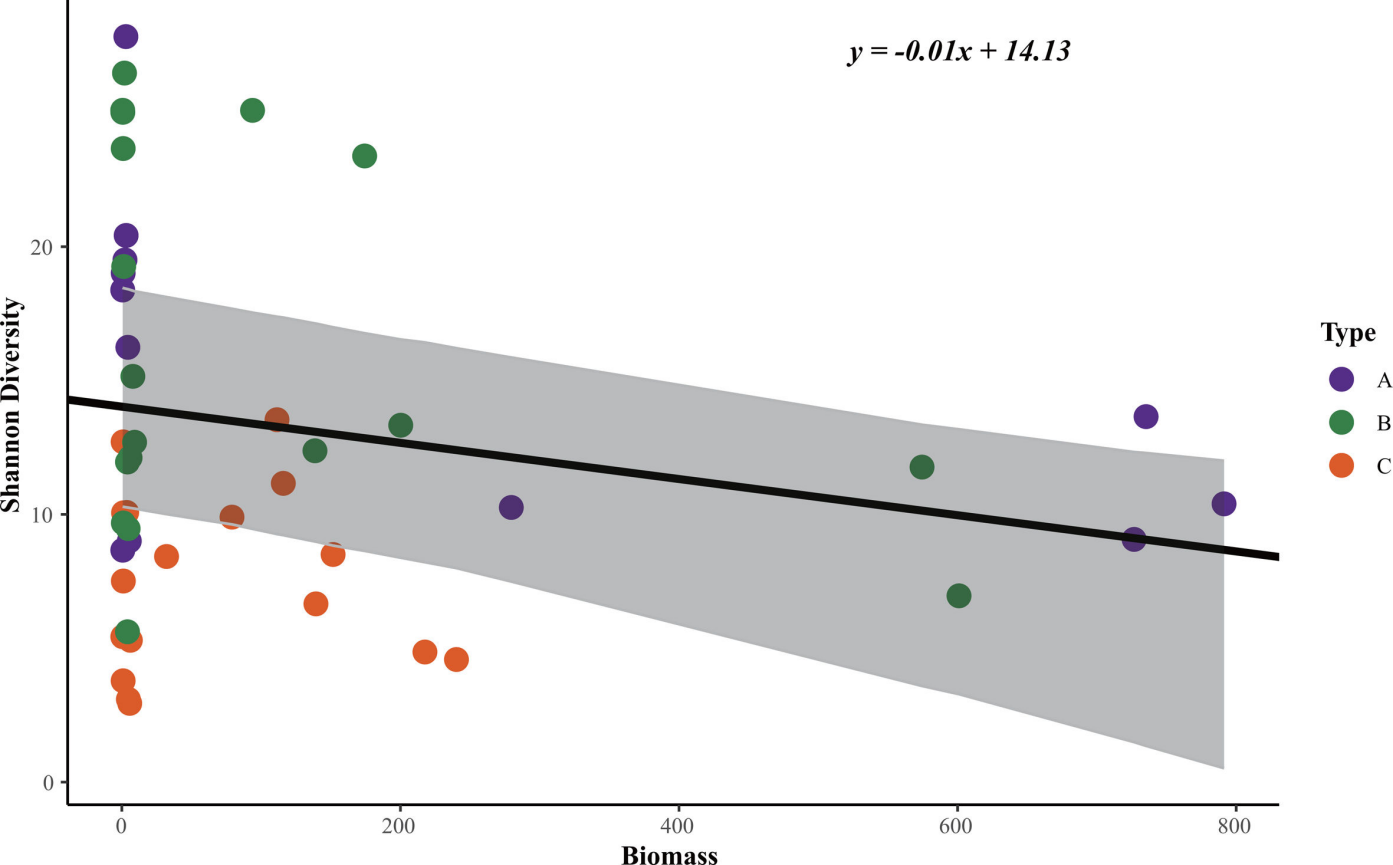
Relationship between phyllosphere pathogen species Shannon diversity and *C. eyrei* aboveground biomass (kg). *P* = 0.02.

The Random Forest approach showed that habitat type was the main factor driving the *α*-diversity of phyllosphere pathogens, regardless of species diversity or phylogenetic diversity; however, age had no significant effect on the *α*-diversity of phyllosphere pathogens (Fig. 3a through c; Table S1). Tree aboveground biomass and diameter at breast height were also important predictors of phyllosphere pathogen and fungal species diversity (Fig. 3b, c, f, and g; Table S1). Therefore, we fitted the relationship between SD, SiD, and *C. eyrei* aboveground biomass with habitat as a random factor and we found a negative relationship (Fig. 4; Fig. S6; Table S2 and S3). Significant effects of both habitat and age were observed only on fungal species richness (Fig. 3e). Leaf dry weight (DW) and leaf organic carbon (LOC) were significant predictors of bacterial species richness (Fig. 3i through l).

**Fig 5:**
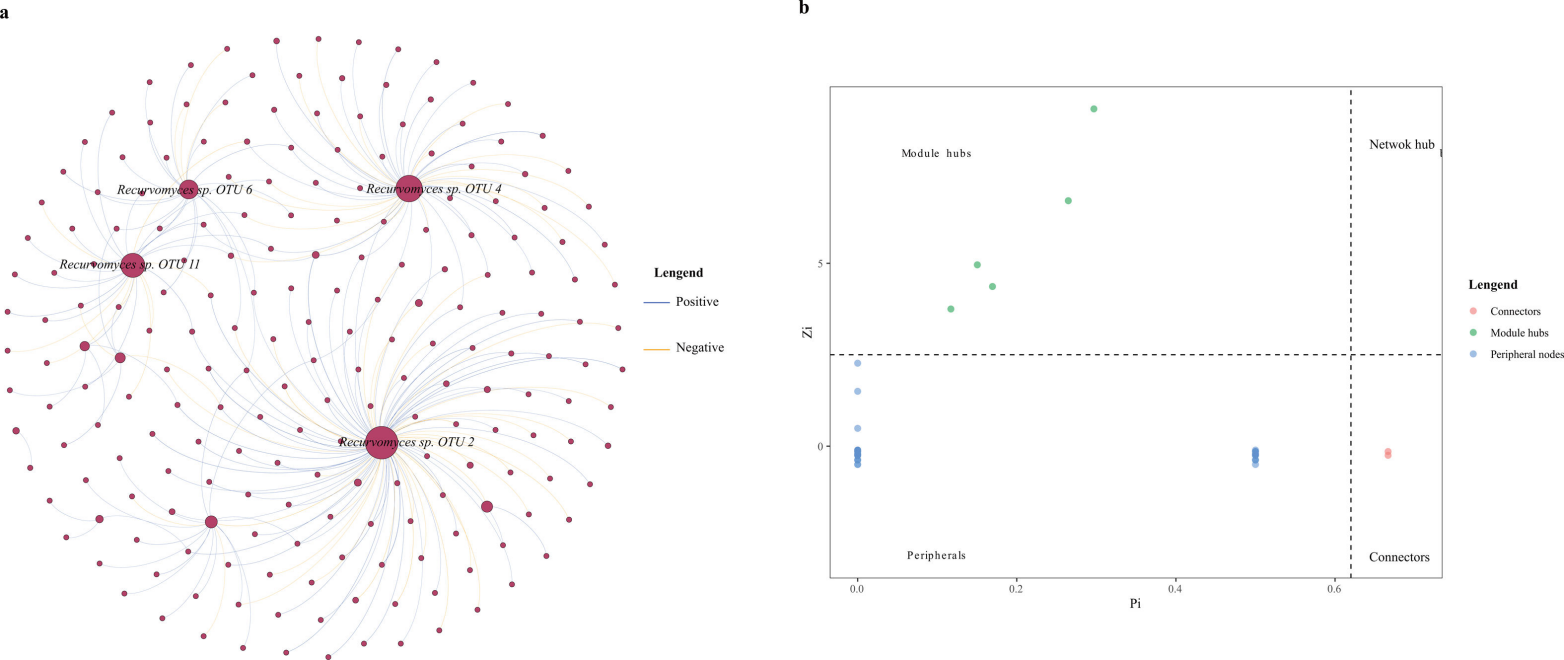
The whole co-occurrence network of pathogen OTUs in the phyllosphere of the *Castanopsis eyrei.* (a) The size of each node represents OTU’s relative abundance. (b) Zi-Pi plots showing the distribution of OTUs with their topological roles in the co-occurrence network of phyllosphere pathogen communities. Green represents the module hubs (Zi >2.5 and Pi <0.62); red represents connectors (Zi <2.5 and Pi >0.62); blue indicates peripherals (Zi <2.5 and pi <0.62).

The results of the co-occurrence network analysis reveal that the network structure for *C. eyrei* saplings is the most complex among the three age stages. However, the network complexity for juveniles and adults is significantly lower than that for saplings and the analysis combining all samples (Table 4). Regardless of the age stage, we identified *Recuromyces* as a potential primary pathogenic fungus colonizing the phyllosphere of *C. eyrei* (Fig. 5a; Fig. S7). *Recuromyces* acts as both a module hub and connector, serving as a key node in this network structure (Fig. 5b).

**TABLE 4:** Topological characteristics of the co-occurrence network of pathogenic fungi in the phyllosphere of *Castanopsis eyrei* at different age stages^*a*^

Attribution	Total	Saplings	Juveniles	Adults
Vertex	213.00	231.00	151.00	179.00
Edge	233.00	258.00	159.00	187.00
Average degree	2.19	2.23	2.11	2.09
Average path length	1.18	1.77	2.12	1.70
Network diameter	2.00	4.00	5.00	3.00
Clustering coefficient	0.003	0.006	0.00	2.56
Density	0.01	0.009	0.01	1.17
Heterogeneity	3.74	2.13	1.66	2.35
Centralization	0.48	0.22	0.22	0.25
Modularity	0.60	0.70	0.72	0.69
Positve	164	146	96	126
Negative	69	112	63	61

^*a*^ Total represents the co-occurrence network analysis considering all samples together.

## DISCUSSION

To the best of our knowledge, we have described in this study the first successful attempt to directly combine bacterial and fungal communities with third-generation high-throughput sequencing to test hypotheses about how phyllosphere fungal and bacterial diversity are maintained across habitats and host plant ages. Moreover, we found that the responses of bacteria and fungi to biotic and abiotic factors were different. Finally, our results showed that phyllosphere pathogens may be a key factor leading to reduced fitness and self-limitation of *C. eyrei*. Below, we discuss the observed patterns and potential mechanisms for the maintenance of phyllosphere microbial diversity.

### The phyllosphere microorganisms of the Fagaceae in the wild exhibit remarkable diversity

The phyllosphere represents a nutrient-poor and stressful environment, which often exhibits relatively low microbial diversity compared with the soil microbiome (24, 39). In contrast to previous studies, that mainly focused on belowground components of the diversity of microbial communities, our research places a primary emphasis on exploring the aboveground microbial diversity. This shift in focus highlights the intricate interplay and mutual influences between aboveground and belowground microbial communities, underscoring their interconnectedness within ecosystems. Our results revealed that the phyllosphere microbes indeed demonstrate lower species diversity and phylogenetic diversity when compared with the soil microbial diversity previously investigated at the same location (40) (Fig. 1). However, at the phylum-level classification, the composition of the phyllosphere microbe communities remains consistent with that of soil microbes (40). One plausible explanation for this pattern is that the phyllosphere microbiota depends heavily on the soil community, with certain endophytic rhizobia present in the soil being capable of migrating to plant leaves through the vascular tissues (41). This migration process likely plays a critical role in shaping the microbial communities in both the aboveground and belowground environments.

In our study, we observed that the phyla Ascomycota and Basidiomycota were prevalent among the phyllosphere fungi of *C. eyrei* (Fig. S4a). This finding is consistent with results from previous research on oak trees (14), where these two phyla were also identified as the dominant fungal groups. Ascomycota and Basidiomycota are among the most diverse and significant of the five major phyla in the fungal kingdom, playing crucial roles in various ecological processes. Ascomycetes, representing approximately 45%–60% of the fungal community (Fig. S4b), emerged as the predominant fungal phylum in our current study. The Ascomycota are known for their great diversity in terms of physiological and ecological characteristics and play a significant role in various ecological processes, particularly in the nitrogen cycle (42). The prevalence of Ascomycetes in the leaf-associated fungal community reflects its crucial involvement in nutrient cycling and ecosystem functioning. Following the Ascomycetes, the Actinobacteria constituted the second most abundant group, accounting for approximately 15%–20% of the fungal community. Actinobacteria have been shown to establish symbiotic relationships with plants, which can exert positive effects on plant growth and health. Their symbiotic association is particularly crucial in facilitating nitrogen supply to the host plant, a process that is fundamental to plant development and ecosystem productivity (43).

In the past, scientific understanding of microbial diversity in the phyllosphere has been limited by the use of second-generation sequencing technologies. These methods provided valuable insights into the overall microbial community composition but had certain limitations in resolving fine-scale taxonomic details and capturing full-length sequences of microbial DNA. In our current study, we employed third-generation full-length sequencing technology to address these limitations and obtain a more comprehensive view of phyllosphere microbial diversity. In our study, we observed that the two most abundant families of fungi in the phyllosphere microbial community were Teratosphaeriaceae and Trimorphomycetaceae. The Teratosphaeriaceae stood out as the predominant family, making up around 16%–31% of the total fungal community. This finding is of significant ecological importance as some genera and species within the Teratosphaeriaceae are known to be plant pathogens, causing important diseases in plants (42, 44). Understanding the prevalence and abundance of these plant pathogenic fungi in the phyllosphere is crucial for gaining insights into their ecological roles and potential impacts on plant health and ecosystem dynamics.

In contrast to the high species diversity observed among the fungi, our study revealed that the species diversity of phyllosphere bacteria was comparatively low, which differed from previous studies, conducted on oaks. Notably, both the temperate zone and laboratory experiments with sterile cultures of oaks exhibited higher phyllosphere bacterial diversity than was obtained from our findings (2, 45). While the exact reason for this difference remains uncertain, several potential explanations warrant consideration. First, the phyllosphere samples used in our study were collected directly from the canopy, differing from seedling samples often used in growth rooms or controlled-environment experiments. This disparity in survival conditions between field-collected samples and those from controlled environments may lead to very different microbial communities. The field conditions in the canopy are more complex and diverse, and the interactions between plants and their microbial partners in such natural settings may significantly influence bacterial diversity (23). Second, there is experimental evidence suggesting a crucial role for carbohydrate and amino acid metabolism in the survival of phyllosphere bacteria (46). Inefficient resource utilization in the canopy environment might be a factor contributing to the observed lower diversity of phyllosphere bacteria than fungi. The availability of resources in natural environments differs significantly from controlled laboratory conditions, which can influence microbial community structures. Furthermore, the sampling sites for our study were located in subtropical forests on Mount Huangshan, while the former controlled-environment study focused on temperate zones (2, 45). Variations in temperature and seasonal changes between the two climatic zones might also contribute to differences in bacterial diversity in the phyllosphere of oak and *C. eyrei* (23, 24). Climatic and environmental factors play a critical role in shaping microbial communities, and such variations can lead to different microbial diversity patterns. In conclusion, despite variations in tree species and climatic zones, the general composition of the phyllosphere microbial community in subtropical forests on Mount Huangshan aligns with that reported in the literature (14, 45, 47), suggesting the existence of a core functional microbiota in the phyllosphere microbial system. Nonetheless, it is essential to recognize the context-specific nature of microbial diversity and consider the complex interactions between trees and their associated microbial communities in natural environments.

### Host habitat determined the phyllosphere microbial community

Our research indicates that habitat, but not tree age, has a significant influence on phyllosphere pathogenic and fungal diversity, both *α*-diversity and *β*-diversity (Tables 1 and 2). This may be because environmental heterogeneity indirectly affects the leaf microbial community of *C. eyrei* by inducing plasticity in plant functional traits that subsequently influence the microbiome (48). However, our findings on bacterial diversity differ from those of Wagner et al. (18), as we did not observe a significant age effect on bacterial diversity but did find such an effect on fungal diversity. This suggests that phyllosphere bacterial diversity may be less responsive to environmental factors than fungal diversity. Several factors may explain these contrasting results. First, the Wagner et al. (18) study was conducted as a controlled-environment experiment, which differs from the complex and dynamic natural environment in which the field-collected leaf samples used in the current study were grown. The diversity of bacteria in the field may be lower than in cultured samples due to various ecological factors. Second, our collected leaf samples were from individuals with a diameter at breast height (DBH) greater than 1 cm, and the stable physiological and biochemical factors within the leaves might contribute to maintaining a relatively stable bacterial community during the growth of the host plant (18). By contrast, fungal diversity might be more influenced by changes in the surrounding habitat. Our multiple regression on distance and matrix (MRM) results support this inference, as we found that leaf physiology had no significant effect on bacterial diversity (Table 3). Lastly, our MRM results showed that the bacterial community structure was influenced by both geographical distance and leaf morphology, suggesting that bacterial communities may be shaped by a combination of stochastic and selective processes. Therefore, it is crucial to exercise caution when identifying the main drivers of the phyllosphere bacterial community assembly at the local scale.

Our findings highlight the significance of habitat type in shaping the accumulation of phytopathogenic fungi and other fungi in the phyllosphere (Fig. 3a through c). One possible explanation for this observation is that different habitats exhibit variations in soil and microclimate conditions, influencing fungal communities and consequently impacting the relationship between fungi and plant assemblages, ultimately influencing plant community composition in different habitats (49, 50). Interestingly, we also identified the diameter at breast height and tree biomass to be important factors influencing phytopathogenic fungal community composition. Moreover, we observed a negative correlation between aboveground tree biomass and phytopathogenic fungal diversity (Fig. 3b, c and 4). Our findings suggest that, in the presence of leaf pathogenic fungi, phyllosphere pathogenic fungal diversity tends to decrease with increasing host biomass. This may be due to the larger tree individuals being probably older and more prone to self-limitation, leading to a dilution effect of plant species identity or neighboring individual traits on foliar pathogen diversity (51). An experimental study in a grassland ecosystem showed support for conspecific negative density dependence (CNDD) mediated by foliar fungal pathogens and enemy-mediated facilitation, promoting species coexistence and structuring plant communities (7). This new finding highlights the potential role of phyllosphere pathogens in causing negative density dependence for a population independent of other populations within subtropical forests. The complexity of symbiotic network structures is often used to infer the strength of interactions among fungi (52). To further validate the possible presence of CNDD, our co-occurrence network analysis revealed that the pathogenic fungal network structure of saplings is more stable (Table 3). Regardless of host age, *Recuromyces* consistently emerged as a core pathogenic fungus. From this, we can infer the following conclusions: first, saplings are more susceptible to colonization by pathogens compared to adult trees and juveniles. Second, epiphytic pathogens may exhibit host specificity, which is a key assumption of the Janzen-Connell effect (8, 9).

Furthermore, we found that bacterial diversity was more responsive to leaf dry weight (DW). This may be attributed to the fact that plants (primary producers) provide organic carbon essential for decomposers (53). A relationship between plant productivity and microbial biomass has previously been suggested (54, 55), indicating a crucial role for leaf dry matter content in shaping microbial community assembly. However, it is important to note that our analysis focused solely on the relationship between biomass and pathogen diversity and provided only a partial explanation for how phyllosphere pathogenic fungi mediate species coexistence in subtropical forests. In future research, we aim to gain a more comprehensive understanding of the feedback between different phyllosphere fungal guilds and the CNDD of subtropical forest tree species in Mount Huangshan. This approach will offer deeper insights into the complex ecological interactions that influence plant community dynamics in the subtropical forest ecosystem.

### Conclusion

Our findings carry significant implications for understanding the hyperdiverse phyllosphere microbial communities and their mechanisms for diversity maintenance in subtropical forests. Notably, we underlined the pivotal role of habitat as a crucial abiotic determinant influencing the presence of phyllosphere fungi and pathogens, compared with the age factor. Our study revealed notable variations in the relative abundance of phyllosphere fungi across different habitats, discernible even at the taxonomic levels of phylum and family. Furthermore, we highlighted the potential divergence in the study of phyllosphere microbes within distinct habitats, particularly when studying at finer taxonomic resolutions. In addition, our research showed the influence of intrinsic host characteristics on phyllosphere fungal diversity. We highlighted how the host’s biomass, DBH, and pH can have an impact on the maintenance of the intricate web of phyllosphere fungal diversity. This intriguing pattern of potential negative correlations introduces a fresh perspective into the realm of negative density dependence on plant community dynamics. Moreover, in the context of bacterial diversity, we established that phyllosphere bacterial diversity is relatively low and appears to be predominantly influenced by leaf dry weight. This multifaceted exploration of the interplay between various factors and the composition of phyllosphere microbial communities enriches our understanding of the dynamic interactions within plant–microbe ecosystems.

## Data Availability

Data analyzed in this study are publicly available at Figshare (https://figshare.com/articles/dataset/Untitled_Item/24113325).

## SUPPLEMENTAL MATERIAL

Supplemental figures and tables spectrum.02091-24-s0001.docx Fig. S1 to S7; Tables S1 to S3.

ASM does not own the copyrights to Supplemental Material that may be linked to, or accessed through, an article. The authors have granted ASM a non-exclusive, world-wide license to publish the Supplemental Material files. Please contact the corresponding author directly for reuse.
