# Erratum for Xie et al “The effects of small geographical resolution and age on the phyllosphere microbial diversity of *Castanopsis eyrei* in subtropical forest”

**DOI:** 10.1128/spectrum.01670-25

**Published:** 2025-08-27

**Authors:** Lei Xie, XuXu Bao, Shuifei Chen, Hui Ding, Yanming Fang

## ERRATUM

Volume 13, no. 3, e02091-24, 2025, https://doi.org/10.1128/spectrum.02091-24: “*Recurvomyces*” should be spelled consistently throughout the manuscript as shown in this erratum.

