# Supplementary material for: The effects of small geographical resolution and age on the phyllosphere microbial diversity of Castanopsis eyrei in subtropical forest
Source: Microbiol Spectr. 2025 Feb 12;13(3):e02091-24. doi: 10.1128/spectrum.02091-24 (PMC11878032; doi:10.1128/spectrum.02091-24)
Supplement: Supplemental figures and tables — Fig. S1 to S7; Tables S1 to S3. [file spectrum.02091-24-s0001.docx]

*Supplementary Information for*

The effects of small geographical resolution and age on the phyllosphere microbial diversity of *Castanopsis eyrei* in subtropical forest

**This file includes:**

Fig. S1 – Fig. S7

Table S1-S3


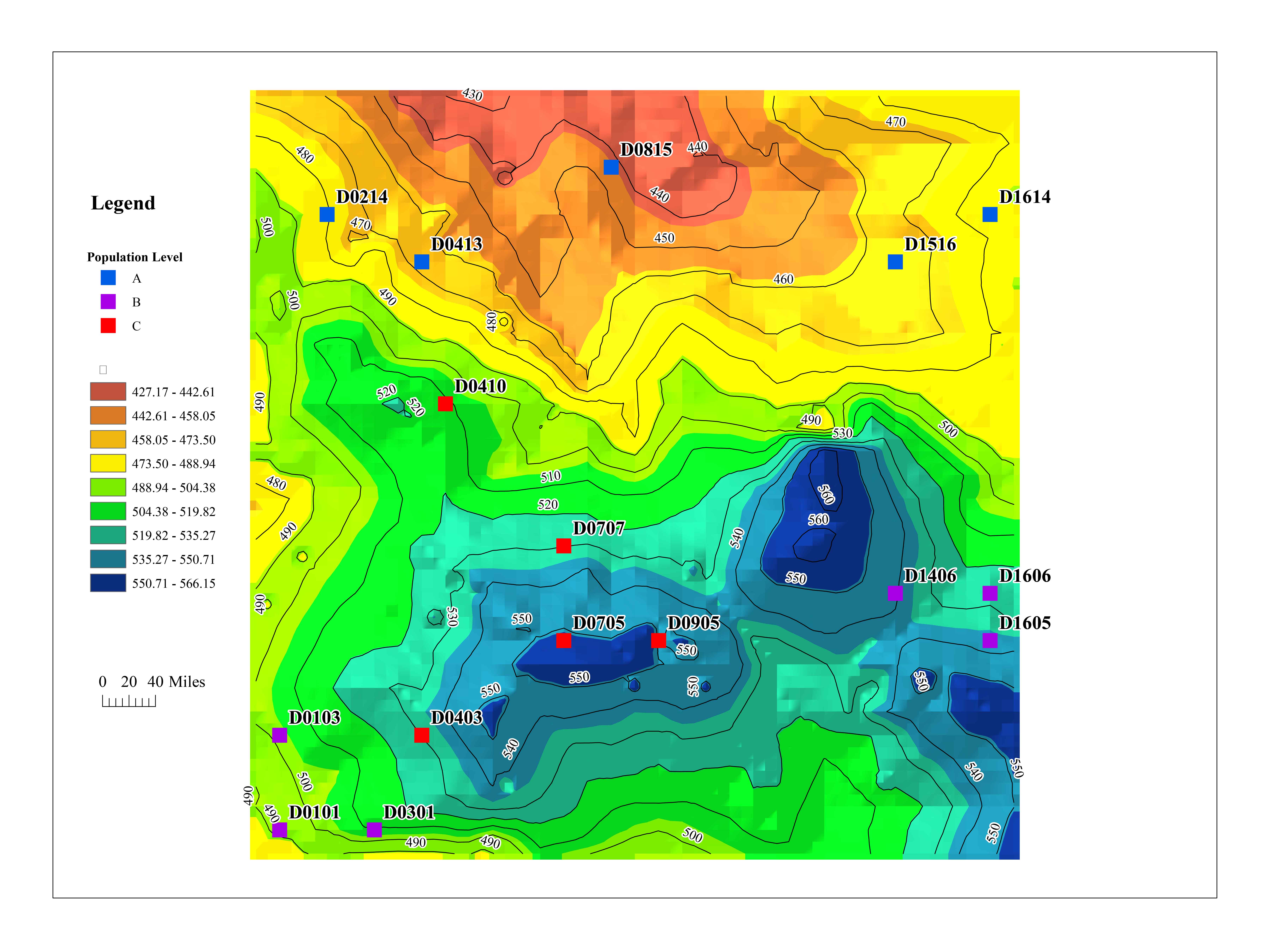


**Fig. S1** Distribution of 16 quadrats sampled in this study in 10.24 hm^2^ forests dynamics plot in Mt. Huangshan. Different colours indicate that phyllosphere samples were collected in different habitats. The labels in the figure denote the names of each 20 × 20 m quadrats.


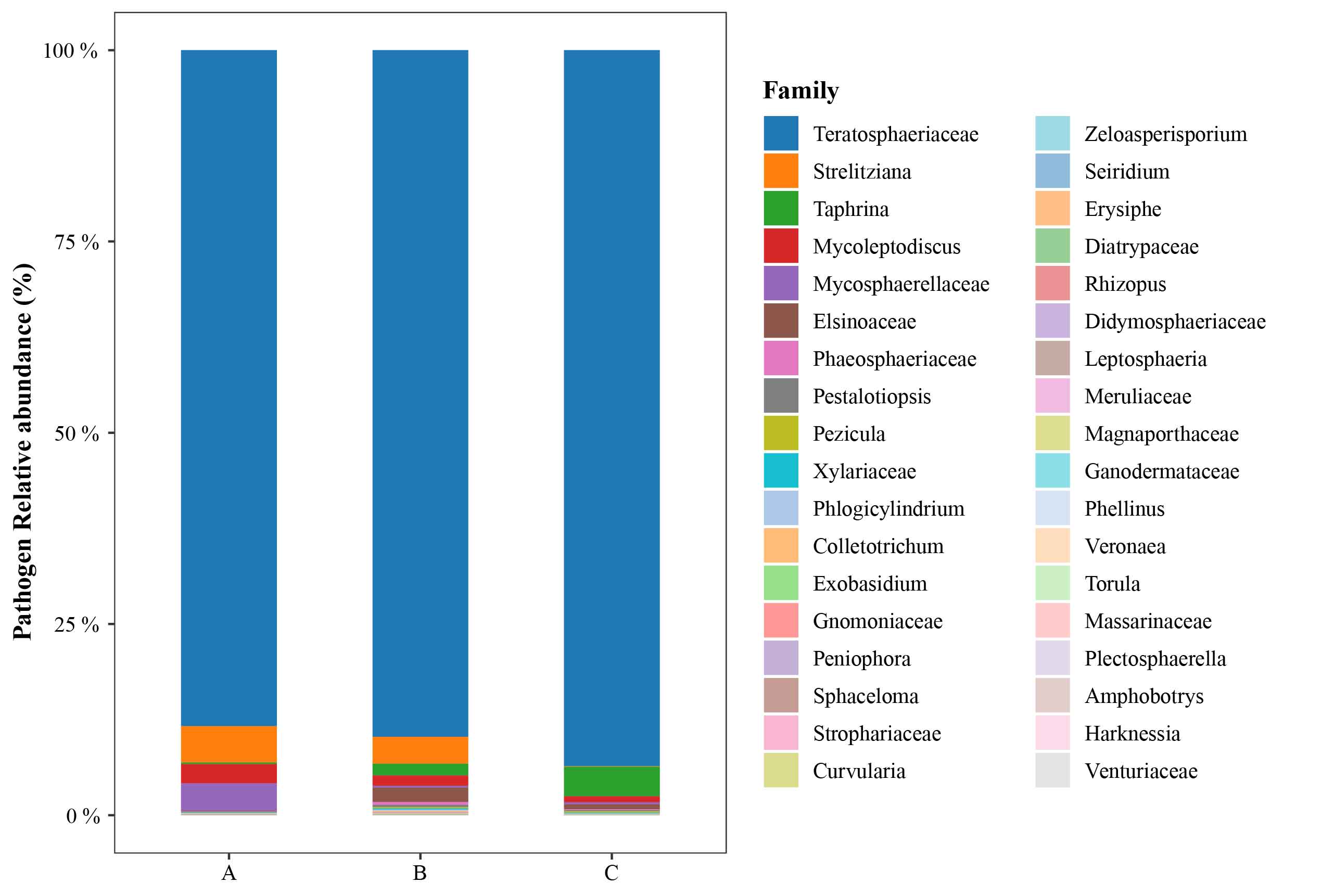


**Fig. S2** Main taxonomic compositions of the phyllosphere of *C. eyrei* pathogen communities in three habitats.


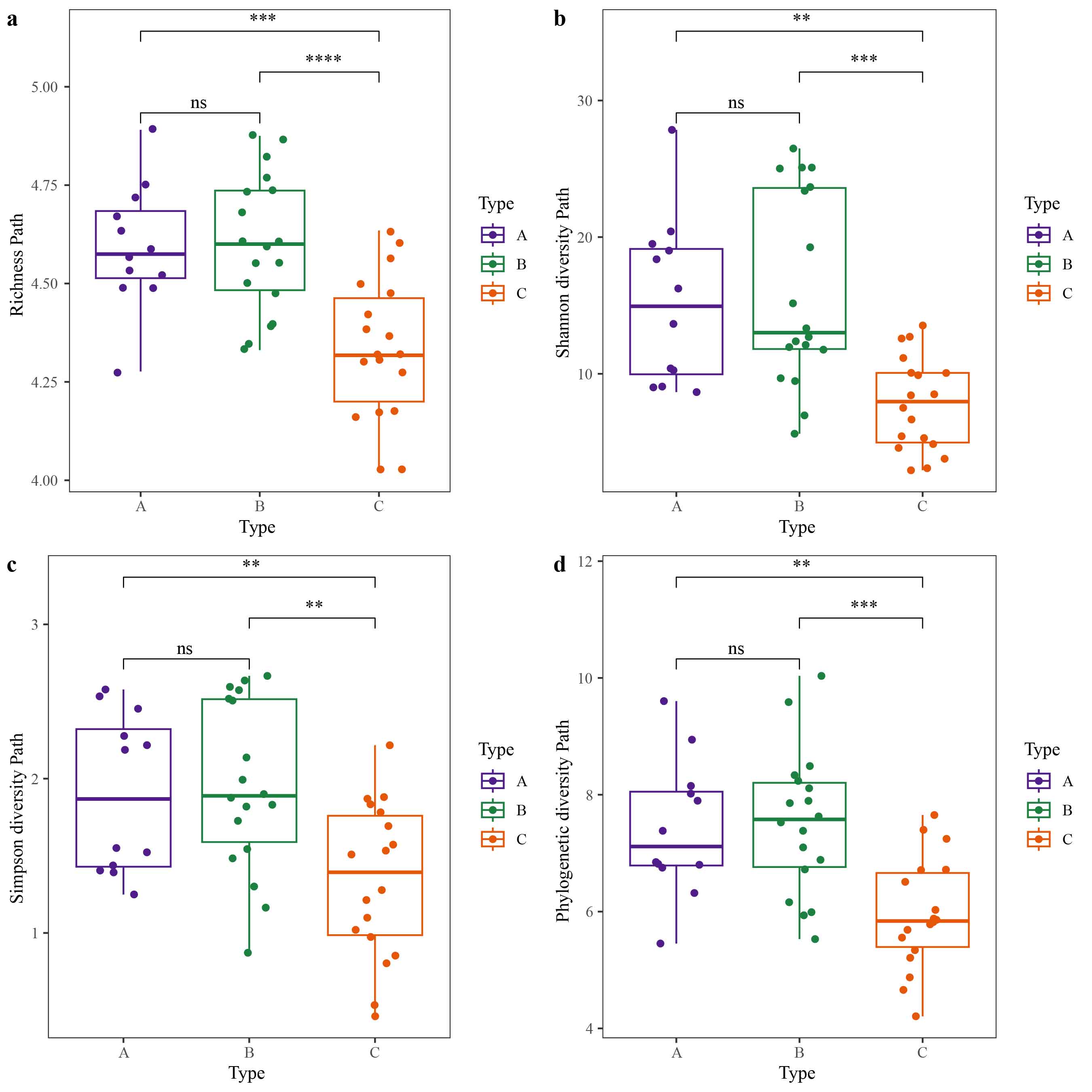


**Fig. S3** The observed OTU richness, Shannon diversity, Simpson diversity and phylogenetic diversity for pathogen.


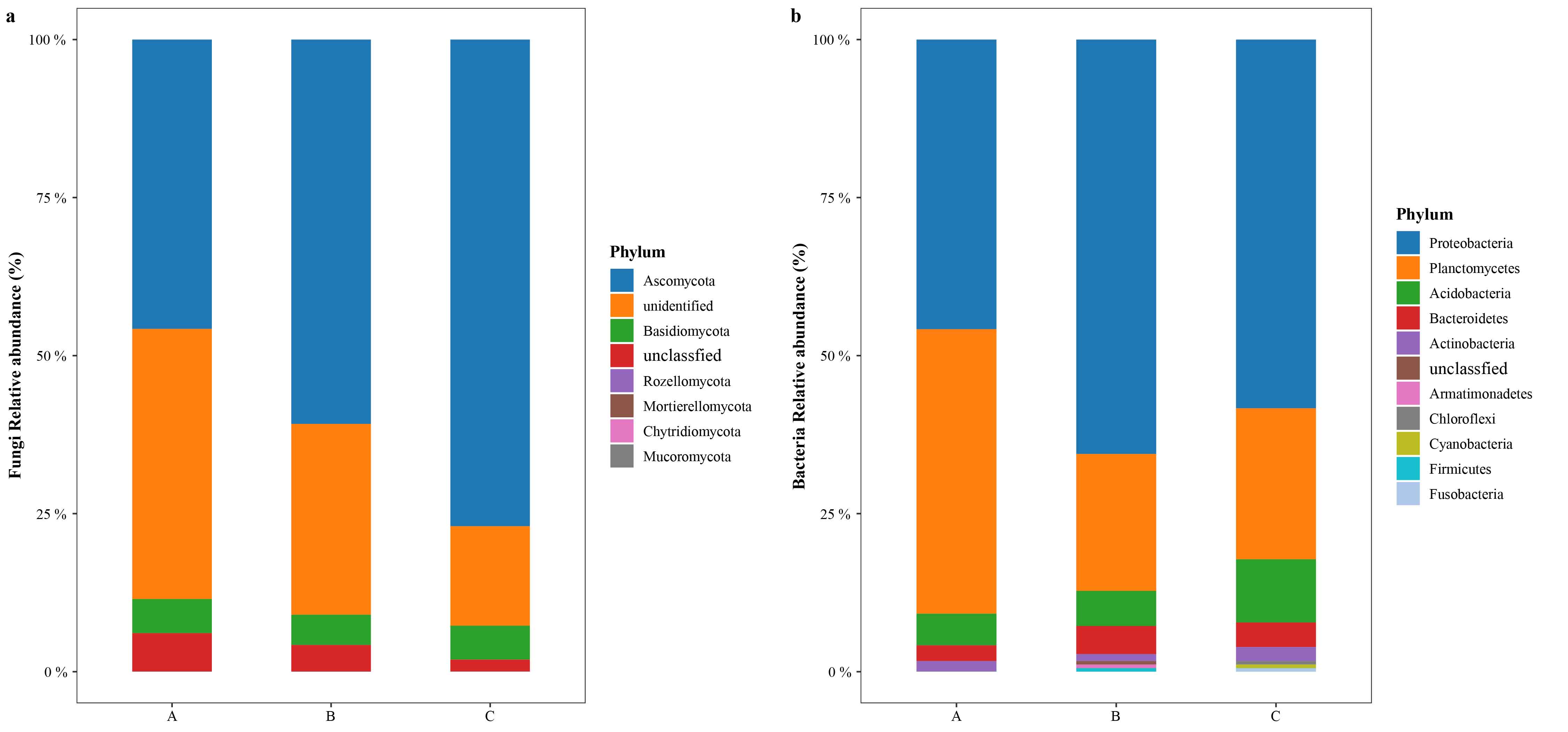


**Fig. S4** Main taxonomic compositions of the phyllosphere of *C. eyrei* fungal (a) and bacterial (b) communities in three habitats.

**
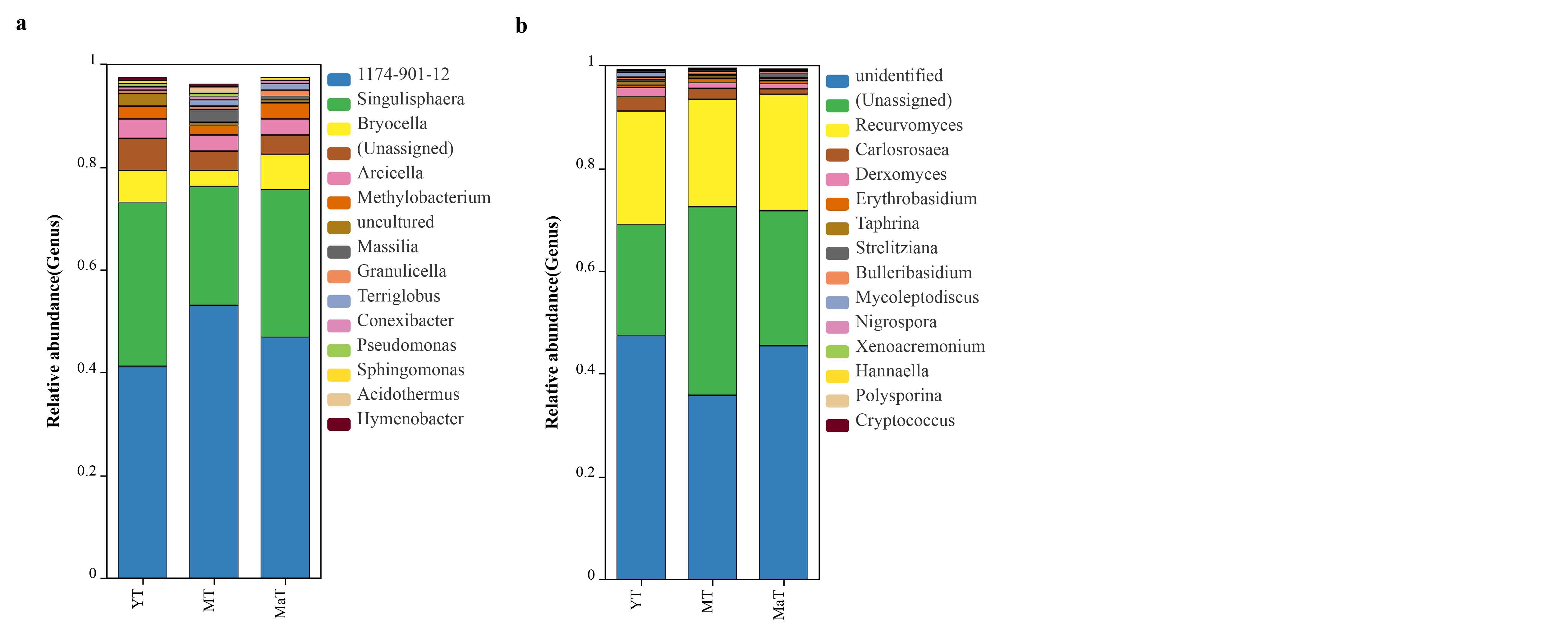
Fig. S5** Main taxonomic compositions of the phyllosphere of *C. eyrei* fungal (a) and bacterial (b) communities in three habitats.

**
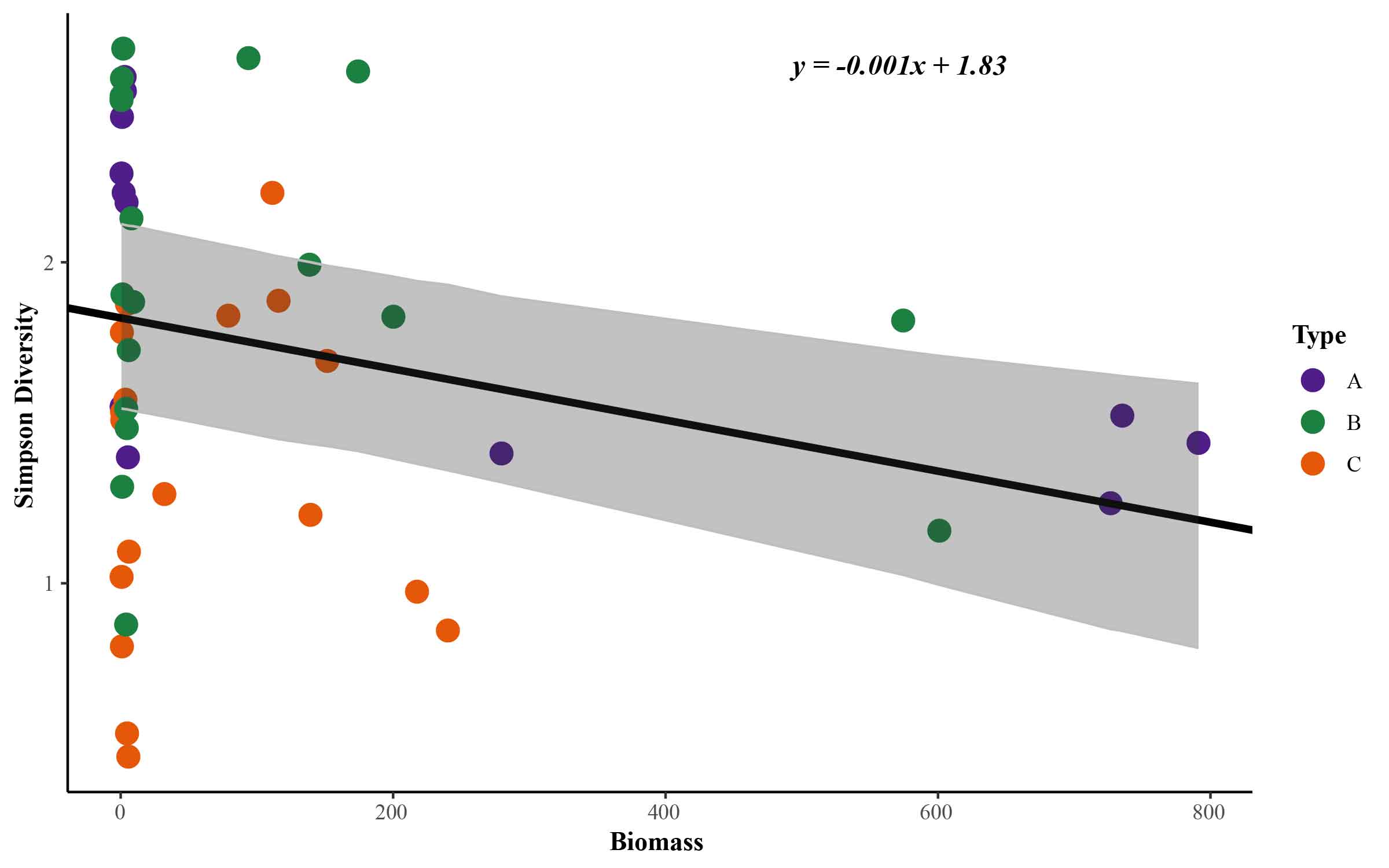
**

**Fig. S6** Relationship between plant pathogen Simpson diversity and biomass. *p* = 0.03.





**Fig. S7** The co-occurrence network of fungal OTUs in the phyllosphere of the different stages of *Castanopsis eyrei* (a) Saplings (YT); (b) Juveniles (MT); (c) Adults (MAT).

**Table. S1** R^2^ for the final RF model

| RF model | R^2^ (%) |
| --- | --- |
| a. Pathogenic Richness~ Habitat Type + Convexity | 26.06 |
| b. Pathogenic Simpson Diversity ~ Habitat Type + DBH + Biomass | 13.75 |
| c. Pathogenic Shannon Diversity ~ Habitat Type + DBH + Biomass | 32.05 |
| d. Pathogenic Phylogenetic Diversity ~ Habitat Type | 1.55 |
| e. Fungal Richness ~ Habitat Type + Age | 32.26 |
| f. Fungal Simpson Diversity ~ DBH + pH | 0 |
| g. Fungal Shannon Diversity ~ Biomass + DBH + Slope | 0 |
| h. Fungal Phylogenetic Diversity ~ Habitat Type + Drought weight | 33.6 |
| i. Bacterial Richness ~ Drought weight | 3.51 |
| g. Bacterial Simpson Diversity ~ | 0 |
| k. Bacterial Shannon Diversity ~ Drought weight | 0 |
| i. Bacterial Phylogenetic Diversity ~ Organic carbon | 0 |

Note: Significant effects are list in the table.

**Table. S2** Results of linear mixed-effects revealing the relationships between phyllosphere pathogen shannon diversity and biomass

| Predictors | Estimates | CI | *p* |
| --- | --- | --- | --- |
| Intercept | 14.13 | 8.2-20.05 | < 0.001 |
| Biomass | -0.01 | -0.02-0.00 | 0.024 |
| Randoms Effects | | | |
| σ^2^ | 28.79 | | |
| τ_00Habitat_ | 23.40 | | |
| ICC | 0.45 | | |
| N_Habitat_ | 3 | | |

Note: Marginal R^2^ / Conditional R^2^ = 0.065 / 0.484

**Table. S3** Results of linear mixed-effects revealing the relationships between phyllosphere pathogen simpson diversity and biomass

| Predictors | Estimates | CI | *p* |
| --- | --- | --- | --- |
| Intercept | 1.83 | 8.2-20.05 | < 0.001 |
| Biomass | -0.80e^-3^ | -0.02-0.00 | 0.031 |
| Randoms Effects | | | |
| σ^2^ | 0.25 | | |
| τ_00Habitat_ | 0.13 | | |
| ICC | 0.35 | | |
| N_Habitat_ | 3 | | |

Note: Marginal R^2^ / Conditional R^2^ = 0.069 / 0.393
